# Cyclin-dependent kinase 9 (CDK9) predicts recurrence in Middle Eastern epithelial ovarian cancer

**DOI:** 10.1186/s13048-021-00827-8

**Published:** 2021-05-20

**Authors:** Sandeep Kumar Parvathareddy, Abdul K. Siraj, Tariq Masoodi, Padmanaban Annaiyappanaidu, Ismail A. Al-Badawi, Fouad Al-Dayel, Khawla S. Al-Kuraya

**Affiliations:** 1 Human Cancer Genomic Research, King Faisal Specialist Hospital and Research Center, P.O. Box 3354, MBC#98 − 16, 11211 Riyadh, Saudi Arabia; 2 Department of Obstetrics & Gynecology, King Faisal Specialist Hospital and Research Centre, Riyadh, Saudi Arabia; 3 Department of Pathology, King Faisal Specialist Hospital and Research Centre, P.O. Box 3354, 11211 Riyadh, Saudi Arabia

**Keywords:** Cyclin-dependent kinase, Epithelial ovarian cancer, Recurrence, Immunohistochemistry

## Abstract

**Background:**

Cyclin-dependent kinase 9 (CDK9) has been shown to play an important role in tumorigenesis of several malignancies. However, the expression of CDK9 in ovarian cancer from Middle Eastern ethnicity remains unknown.

**Methods:**

A tissue microarray of 441 epithelial ovarian cancer (EOC) samples was used to study the expression of CDK9 immunohistochemically and their clinico-pathological associations were determined. Cox proportional hazards regression model was used for univariate and multivariate analysis of recurrence-free survival.

**Results:**

CDK9 over-expression was noted in 56.2 % (248/441) of EOCs and was associated with adverse clinico-pathological parameters such as distant metastasis (*p* < 0.0001), stage IV tumors (*p* < 0.0001), tumor recurrence (*p* = 0.0105) and high Ki-67 index (*p* < 0.0001). Importantly, CDK9 over-expression was an independent predictor of poor recurrence-free survival (Hazard ratio = 1.51; 95 % confidence interval = 1.15–1.98; *p* = 0.0030). We also found that CDK9 outperforms Ki-67 as a predictor of tumor recurrence in EOC.

**Conclusions:**

Our results show that CDK9 expression correlates with markers of advanced disease in Middle Eastern EOC and is also a prognostic marker. CDK9 overexpression also identifies a subset of patients with highest likelihood of recurrence across the patient cohort. These patients may benefit from additional alternative therapies targeting CKD9.

**Supplementary Information:**

The online version contains supplementary material available at 10.1186/s13048-021-00827-8.

## Background

Today, ovarian cancer (OC) is a major health problem evidenced by the fact that it is the most common cause of death among gynecological malignancies [1, 2]. The majority of OC patients suffer from relapse after standard current therapies and this contributes to the lethality of OC [3–5]. Identifying prognostic biomarkers related to OC recurrence are urgently needed to select patients who might benefit from closer surveillance and more aggressive therapeutic approaches.

Cyclin-dependent kinases (CDKs) are members of heterodimeric serine/thereonine protein kinases and are involved in important cellular functions, like cell cycle progression and DNA transcription [6, 7]. Besides, previous studies have demonstrated that deregulation of CDK and CDK-mediated pathway are associated with progression and tumorigenesis of several cancer entities [8–12]. Thus, CDK inhibitors have been considered as an attractive option for treating a number of human malignancies [13–17].

Recently, cyclin dependent protein kinase 9 (CDK9) has been recognized as a crucial cell cycle regulator and important player in several cancers including ovarian cancer [18–21]. CDK9 and Cyclin T complex are components of the positive transcription elongation factor b (P-TEFb), which helps in release of RNA polymerase into the elongation process [22]. CDK9 is expressed in two isoforms, light 42-kDa isoform and heavy 55-kDa [23]. Both isoforms have been shown to be expressed in human cancer cell lines [24, 25]. Moreover, several reports have demonstrated the prognostic value of CDK9 overexpression in various cancers, including OC [19, 21, 26, 27].

CDK9 prevalence and the relationship between its expression and clinical prognosis in Middle Eastern OC patients remains to be elucidated. Therefore, we conducted this study to evaluate the incidence of CDK9 overexpression in Middle Eastern epithelial ovarian cancer (EOC) patient specimens and explore its predictive as well as prognostic value in this patient cohort.

## Materials and methods

### Sample selection

Archival samples from 441 EOC patients diagnosed between 1989 and 2017 at King Faisal Specialist Hospital and Research Center (Riyadh, Saudi Arabia) were included in the study. Corresponding local (peritoneal) metastatic and distant metastatic tissues were available for 194 and 9 cases, respectively. Primary tumor samples and the corresponding peritoneal metastases were collected at the same time for each patient. Detailed clinico-pathological data were noted from case records and have been summarized in Table 1. Recurrence-free survival was computed from date of surgery for patients who underwent primary surgery to date of disease progression or recurrence (local, regional or distant). The median follow-up time was 20 months (range, 2–349 months). Tumors were classified according to WHO Classification of female genital tumors (2020). International Federation of Gynecology and Obstetrics (FIGO) system was used for staging and grading of tumors.

**Table 1:** Clinicopathological variables for the patient cohort (*n* = 441)

	n (%)
**Age**	
Median	50.3
Range	17.0–90.0
**Histopathology**	
High grade Serous	233 (52.8)
Low grade Serous	83 (18.8)
Mucinous	64 (14.5)
Endometrioid	40 (9.1)
Clear cell	10 (2.3)
Undifferentiated	11 (2.5)
**Histological Grade**	
Grade 1	98 (22.2)
Grade 2	152 (34.5)
Grade 3	175 (39.7)
Unknown	16 (3.6)
**pT**	
T1	89 (20.2)
T2	40 (9.1)
T3	307 (69.6)
Unknown	5 (1.1)
**pN**	
N0	401 (90.9)
N1	35 (8.0)
Unknown	5 (1.1)
**pM**	
M0	358 (81.2)
M1	78 (17.7)
Unknown	5 (1.1)
**Stage**	
I	88 (20.0)
II	26 (5.9)
III	244 (55.3)
IV	78 (17.7)
Unknown	5 (1.1)
**Recurrence**	
Yes	145 (32.9)
No	296 (67.1)

All samples were obtained from patients with approval from Institutional Review Board of the hospital. For the study, since only retrospective patient data and archived paraffin tissue blocks were used, a waiver of consent was obtained for the tissue samples, including normal tissue controls, from Research Advisory Council (RAC) under project RAC# 2140033.

### Tissue microarray construction

All samples were analyzed in a tissue microarray (TMA) format. TMA construction was performed as described earlier [28]. Briefly, tissue cylinders with a diameter of 0.6 mm were punched from representative tumor regions of each donor tissue block and brought into recipient paraffin block using a modified semiautomatic robotic precision instrument (Beecher Instruments, Woodland, WI). Two cores of EOC were arrayed from each case.

### Immunohistochemistry (IHC) staining and evaluation

Standard protocol was followed for manual IHC staining. For antigen retrieval, Dako (Dako Denmark A/S, Glostrup, Denmark) Target Retrieval Solution pH 9.0 (Catalog number S2368) was used, and the slides were placed in Pascal pressure cooker at 120^0^ C for 10 min. The slides were incubated with primary antibody against CDK9 (2316 S, 1:300 dilution, pH 9.0, Cell Signaling Technology, Danvers, Massachusetts, USA). The Dako Envision Plus System kit was used as the secondary detection system with 3, 30-diaminobenzidine as chromogen. All slides were counterstained with hematoxylin, dehydrated, cleared and mounted. Negative controls included omission of the primary antibody. Normal tissues of different organ system were also included in the TMA to serve as control (Supplementary Fig. 1 A & B). Only fresh cut slides were stained simultaneously to minimize the influence of slide aging and maximize reproducibility of the experiment. The slides were independently examined by two pathologists. If there was a discrepancy in the individual scores, both pathologists carried out a re-evaluation until a consensus was reached.

CDK9 immunohistochemical expression was seen in the nuclear compartment and quantified using the proportion score, as described previously [21]. Briefly, the proportion of positively stained tumor cells was calculated as a percentage for each core and the scores were averaged across two tissue cores from the same tumor to yield a single percent staining score representing each cancer patient. Cases showing expression level of more than 25 % were classified as overexpression and those with ≤ 25 % as low expression.

Staining and scoring of Ki-67 was performed as described previously, using H score [29]. Briefly, each TMA spot was assigned an intensity score from 0 to 3 (I0, I1-3) and proportion of the tumor staining for that intensity was recorded from a range of 0-100 (P0, P1-3). P0 and P1-3 signify the proportion of positively stained cells corresponding to the respective intensity score (I0, I1-3) (i.e., P1 signifies the proportion of tumor cells which show intensity I1, P2 signifies the proportion of tumor cells which show intensity I2, and so on). A final H score (range 0-300) was obtained by adding the sum of scores obtained for each intensity (I) and proportion (P) of area stained (H score = I1XP1 + I2XP2 + I3XP3). Two scores per tumor were analyzed in order to minimize the number of missing/un-interpretable spots. However, the higher of the two scores was used as the final score. X-tile plots were constructed for assessment of biomarker and optimization of cut-off points based on outcome, as has been described earlier [30]. Based on X-tile plots, EOC cases were classified into two subgroups: those with H score ≤ 50 were defined as low expression of Ki-67 and those with H score > 50 were defined as high expression.

### Statistical analysis

The associations between clinico-pathological variables and protein expression was performed using contingency table analysis and Chi square tests. Mantel-Cox log-rank test was used to evaluate recurrence-free survival. Survival curves were generated using the Kaplan-Meier method. Cox proportional hazards regression model was used for univariate and multivariate analysis. The multivariable Cox proportional hazards models for each cohort were initially fit with the following covariates: age, tumor grade, T stage, nodal status, tumor stage and CDK9 expression status. The final multivariable Cox models were selected using backward elimination procedure. Two-sided tests were used for statistical analyses with a limit of significance defined as *p* value < 0.05. Chi-square test, Mantel-Cox log rank test and survival curve were generated using the JMP14.0 (SAS Institute, Inc., Cary, NC) software package. Evaluation of variable importance in the model was performed using R version 4.0.1 with libraries survival and rms. Model evaluation was plotted using nomogram.

## Results

### Patient characteristics

Median age of the study population was 50.3 years (range: 17–90 years). Majority of the cases were high grade serous carcinoma (52.8 %), followed by low grade serous (18.8 %) and mucinous (14.5 %) carcinomas. The tumors were predominantly grade 2 or 3 (74.2 %) and stage III (55.3 %). 8 % (35/441) had lymph node metastasis and 17.7 % (78/441) had distant metastasis at the time of diagnosis. 32.9 % (145/441) of patients developed tumor recurrence during follow-up (Table 1).

### CDK9 expression and its clinico-pathological associations

**Fig. 1:**
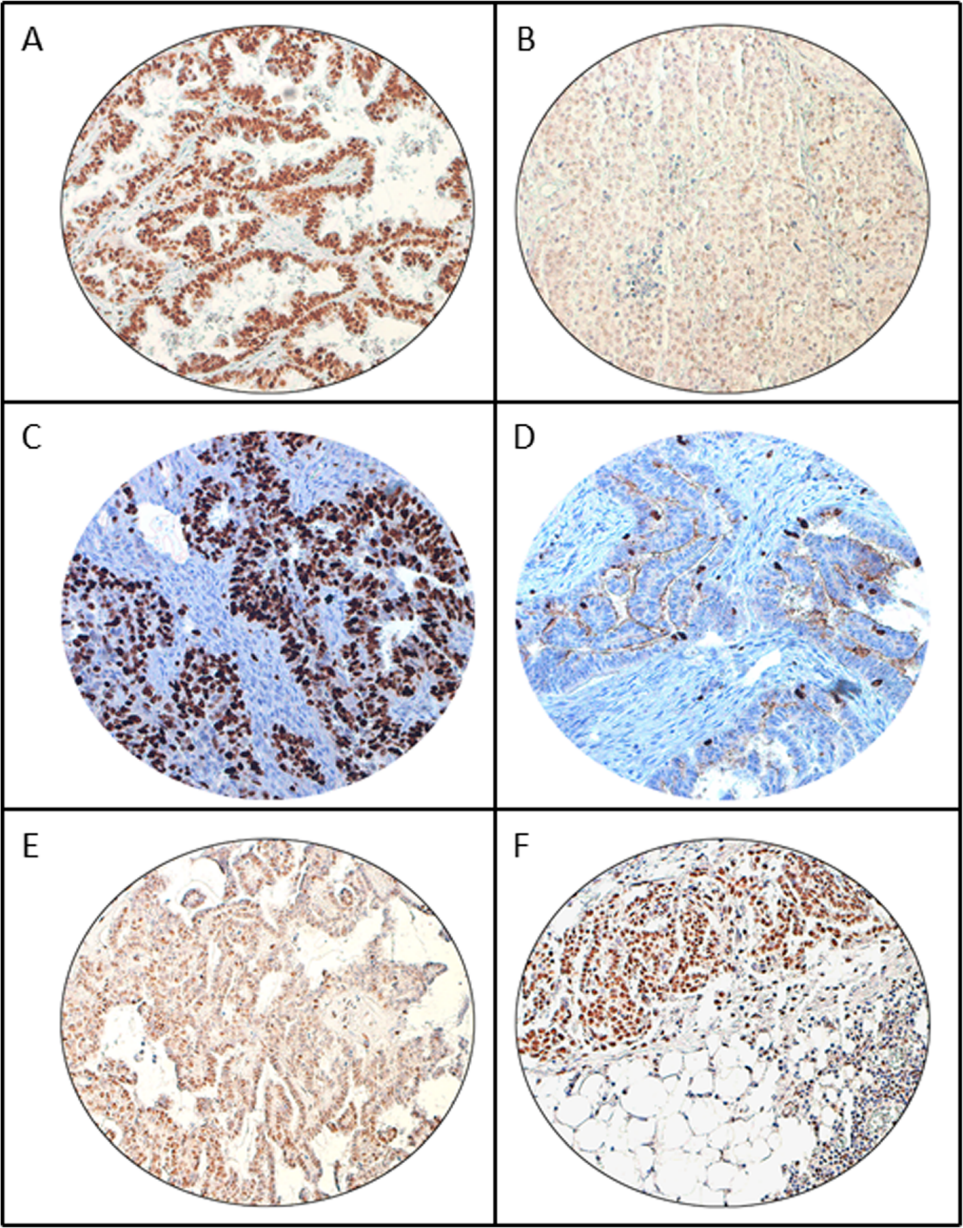
Immunohistochemical analysis of CDK9 expression in primary epithelial ovarian cancer (EOC) and corresponding metastatic tissue. EOC array spots showing **a** over-expression and **b** low expression of CDK9 in primary tumor, **c** over-expression and **d** low expression of Ki-67 in primary tumor. **e** low nuclear expression of CDK9 and **f** high nuclear expression of CDK9 in metastatic tissue. 20X/0.70 objective on an Olympus BX 51 microscope. (Olympus America Inc, Center Valley, PA, USA)

CDK9 was primarily expressed in the nucleus (Fig. 1). CDK9 over-expression was noted in 56.2 % (248/441) of EOCs and was significantly associated with adverse clinico-pathological parameters such as distant metastasis (*p* < 0.0001), stage IV tumors (*p* < 0.0001), tumor recurrence (*p* = 0.0105) and high Ki-67 index (*p* < 0.0001) (Table 2). We also examined the immunohistochemical expression of CDK9 in 4 ovarian cancer cell lines (SKOV-3, OVSAHO, OVCAR-3 and COLO-704) and found that it was over-expressed in all these cell lines (Supplementary Fig. 1 C & D).

**Table 2:** Clinicopathological associations of CDK9 protein expression in EOC

	Total		CDK9 overexpression		CDK9 low expression		*p* value
	**No.**	**%**	**No.**	**%**	**No.**	**%**	
**No. of patients**	441		248	56.2	193	43.8	
**Age (Yrs)**							
≤ 50	221	50.1	129	58.4	92	41.6	0.3649
> 50	220	49.9	119	54.1	101	45.9	
**Histology Type**							
High grade Serous	233	52.8	146	62.7	87	37.3	0.0623
Low grade Serous	83	18.8	37	44.6	46	55.4	
Mucinous	64	14.5	31	48.4	33	51.6	
Endometrioid	40	9.1	22	55.0	18	45.0	
Clear cell	10	2.3	5	50.0	5	50.0	
Undifferentiated	11	2.5	7	63.6	4	36.4	
**Histological Grade**							
Grade 1	98	23.1	46	46.9	52	53.1	0.0758
Grade 2	152	35.8	91	59.9	61	40.1	
Grade 3	175	41.2	105	60.0	70	40.0	
**pT**							
T1	89	20.4	52	58.4	37	41.6	0.7125
T2	40	9.2	24	60.0	16	40.0	
T3	307	70.4	168	54.7	139	45.3	
**pN**							
pN0	401	92.0	225	56.1	176	43.9	0.8351
pN1	35	8.0	19	54.3	16	45.7	
**pM**							
pM0	358	82.1	181	50.6	177	49.4	< 0.0001
pM1	78	17.9	63	80.8	15	19.2	
**Stage**							
I	89	20.4	52	58.4	37	41.6	< 0.0001
II	26	5.9	11	42.3	15	57.7	
III	244	55.8	119	48.8	125	51.2	
IV	78	17.9	63	80.8	15	19.2	
**Recurrence**							
Yes	145	32.9	94	64.8	51	35.2	0.0105
No	296	67.1	154	52.0	142	48.0	
**Ki-67**							
High	218	51.5	152	69.7	66	30.3	< 0.0001
Low	205	48.5	88	42.9	117	57.1	

We next sought to analyse the expression status of CDK9 in metastatic tissues and compare it with the expression in primary EOCs. Local (peritoneal) and distant metastatic tissues were available for 203 EOC cases and the incidence of CDK9 over-expression in these metastatic tissues was significantly higher when compared to their corresponding primary EOC (73.4 % vs. 57.6 %, *p* = 0.0008) (Table 3).

**Table 3:** Comparison of CDK9 expression between primary epithelial ovarian cancer and corresponding metastatic tissues

CDK9	Paired metastatic tissue			*p* value
	High	Low	Total	
**Primary tumor**				
High	92	25	117 (57.6 %)	
Low	57	29	86 (42.4 %)	
Total	149 (73.4 %)	54 (26.6 %)	203	0.0008

### CDK9 expression is associated with poor prognosis

**Fig. 2:**
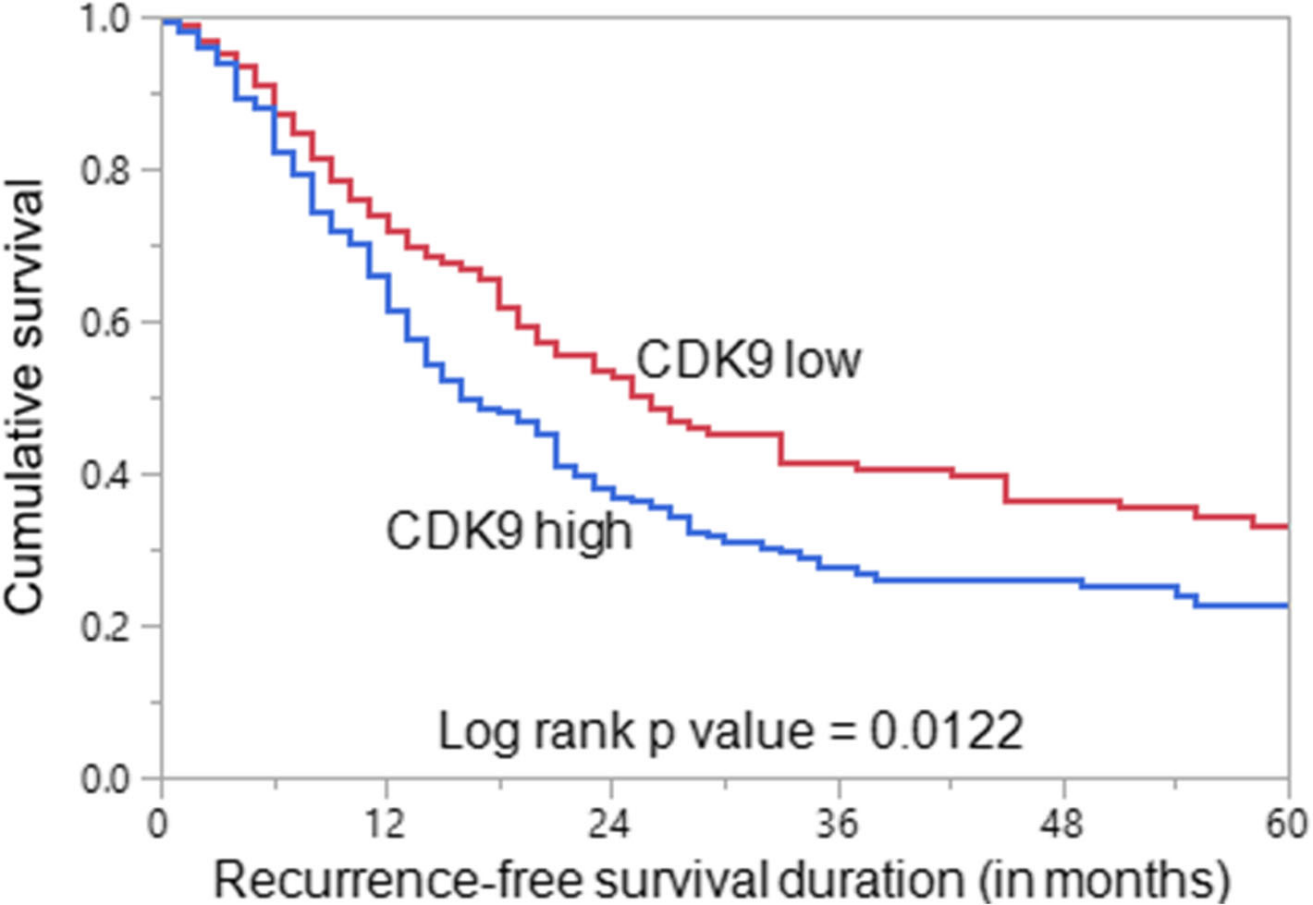
Survival analysis of CDK9 protein expression in epithelial ovarian cancer (EOC). Kaplan-Meier survival analysis for the prognostic significance of CDK9 expression in EOC showed that patients with overexpression of CDK9 had reduced recurrence-free survival at 5 years compared to tumors showing low expression of CDK9 (*p* = 0.0122)

CDK9 over-expression was significantly associated with poor recurrence-free survival (RFS) (*p* = 0.0122) in our cohort (Fig. 2). The median RFS for patients over-expressing CDK9 was 12 months compared to 20 months for those showing low expression of CDK9. On multivariate analysis using Cox proportional hazards model with backward elimination, we found CDK9 to be an independent predictor of poor RFS (Hazard ratio = 1.51; 95 % confidence interval = 1.15–1.98; *p* = 0.0030) (Table 4).

**Table 4:** Univariate and multivariate analysis of clinico-pathological variables and CDK9 expression using Cox proportional hazard model for recurrence-free survival

	Recurrence-free survival			
	Univariate		Multivariate	
Clinico-pathological variables	Hazard ratio (95 % CI)	*p*-value	Hazard ratio (95 % CI)	*p*-value
**Age (years)** > 50 (vs. ≤ 50)	1.51 (1.17–1.94)	0.0013	1.33 (1.02–1.73)	0.0360
**Grade** 3 (vs. 1–2)	1.22 (0.87–1.69)	0.2463		
**pT** T3-4 (vs. T1-2)	0.85 (0.62–1.17)	0.3234		
**Lymph node metastasis** N1 (vs. N0)	1.31 (0.79–2.03)	0.2794		
**Stage** IV (vs. I-III)	2.62 (1.92–3.52)	< 0.0001	3.26 (1.52–6.96)	0.0020
**CDK9** High (vs. Low)	1.38 (1.07–1.79)	0.0132	1.51 (1.15–1.98)	0.0030

### Significance of CDK9 expression from the clinical model with Ki-67

**Fig. 3:**
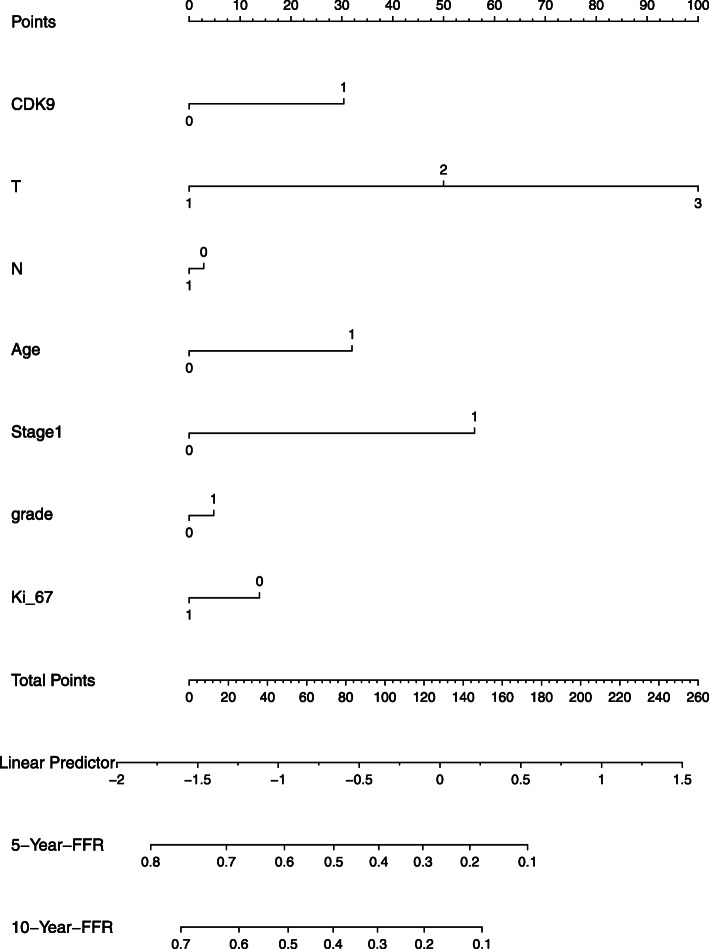
The nomogram of the Cox proportional hazards model with Ki67 in our cohort shows that CDK9 outperforms Ki-67 in predicting recurrence-free survival. RFS – recurrence-free survival

Since we found a significant association between CDK9 and Ki-67 expression, we next sought to determine if the prognostic power of CDK9 persisted even after using the clinical model with Ki-67. The HR of high CDK9 staining compared with low CDK9 staining was 1.44 (95 % CI = 1.06–1.96; *P* = 0.0191), and Ki-67 was not significant at significance level 0.05 (Fig. 3). Our results revealed that CDK9 can separate the freedom from survival plots much more significantly than Ki-67.

## Discussion

Currently, platinum-paclitaxel chemotherapy is given to a large majority of OC patients after cytoreductive surgery [4, 31]. Despite this approach large percentage develop recurrence within 5 years [3, 4, 32]. Identifying predictive markers that can identify OC patients destined for relapse is very valuable since it helps clinicians to offer the use of investigational agents or alternative strategies with existing chemotherapy regimens as initial therapy.

In the present study, we analyzed the expression of CDK9 in 441 Middle Eastern EOC tissue samples. TMA of EOC tissue samples showed that 56 % over-expressed CDK9. A recent study also showed a similar incidence of CDK9 over-expression in OC (50 %), although the sample size was small (*n* = 26, with 24 EOCs) [21]. We have identified a significant association between CDK9 expression and aggressive clinico-pathological parameters such as high proliferative index (Ki-67), advanced stage and tumor recurrence. These findings suggest that CDK9 could be used as a marker of advanced EOC.

Additionally, our study revealed striking correlation between CDK9 levels and RFS. Also, we show that CDK9 is aberrantly expressed in EOC cell lines and that expression of CDK9 in metastatic tissue was significantly higher compared to primary EOC. These data imply the possibility that CDK9 may be related to the progression of EOC in Middle Eastern ethnicity. In concordance with our study, the overexpression of CDK9 and its prognostic value have been documented in several human cancers, including endometrial cancer, chordoma, osteosarcoma, pancreatic cancer and colon cancer [19, 26, 27, 33, 34]. A recent study has demonstrated the effect of CDK9 inhibition on suppression of RNA transcription elongation induction of apoptosis and reduction of proliferation [21]. Although their study on OC tissues from China included only 26 patient samples, they were able to detect the prognostic value of CDK9 overexpression. This is further supportive of the potential role of CDK9 as a promising therapeutic target and demonstrates that selective CDK9 inhibition might be a powerful approach to induce cytotoxic activity in EOC.

Interestingly, our study revealed that CDK9 over-expression was associated with a 1.51-fold increased risk of recurrence in multivariate analysis. To further provide data for the clinical relevance of CDK9 as prognostic marker in Middle Eastern EOC, we interrogated whether CDK9 can outperform Ki-67 in EOC patients. We examined the expression of Ki-67 and compared with CDK9, and the results showed that CDK9 expression can separate the freedom from survival plots more significantly than Ki-67.

Our study demonstrated that CDK9 expression by IHC can be readily assessed at diagnosis and is an important prognostic marker. In addition, these findings suggest that CDK9 may be a potential therapeutic target for EOC treatment. The fact that CDK9 can be readily performed in most pathology laboratories increases the importance of using CDK9 expression as a prognostic and predictive marker. However, further studies are needed to fully illustrate the role of CDK9 in EOC from different ethnic backgrounds.

## Conclusions

In conclusion, our results show that CDK9 expression correlates with markers of advanced disease in Middle Eastern EOC. Furthermore, our result reveal CDK9 as prognostic marker and promising therapeutic target in EOC. CDK9 overexpression also identifies a subset of patients with highest likelihood of recurrence across the patient cohort. These patients may benefit from additional alternative therapies targeting CKD9.

## Supplementary information

**Additional file 1: Figure S1.** CDK9 expression in normal human tissues and ovarian cancer cell lines. CDK9 expression noted in (A) normal kidney, and (B) placenta. CDK9 high expression noted in ovarian cancer cell lines, (C) SKOV-3 and (D) OVCAR-3.

## Data Availability

The datasets used and/or analysed during the current study are available from the corresponding author on reasonable request.

## Abbreviations

CDK: Cyclin-dependent kinase
EOC: Epithelial ovarian cancer
OC: Ovarian cancer
P-TEFb: Positive transcription elongation factor b
WHO: World Health Organization
FIGO: International Federation of Gynecology and Obstetrics
TMA: Tissue microarray
IHC: Immunohistochemistry
RFS: Recurrence-free survival
HR: Hazard ratio
CI: Confidence interval
