# Supplementary figures and images for: Cyclin-dependent kinase 9 (CDK9) predicts recurrence in Middle Eastern epithelial ovarian cancer
Source: J Ovarian Res. 2021 May 20;14:69. doi: 10.1186/s13048-021-00827-8 (PMC8136118; doi:10.1186/s13048-021-00827-8)

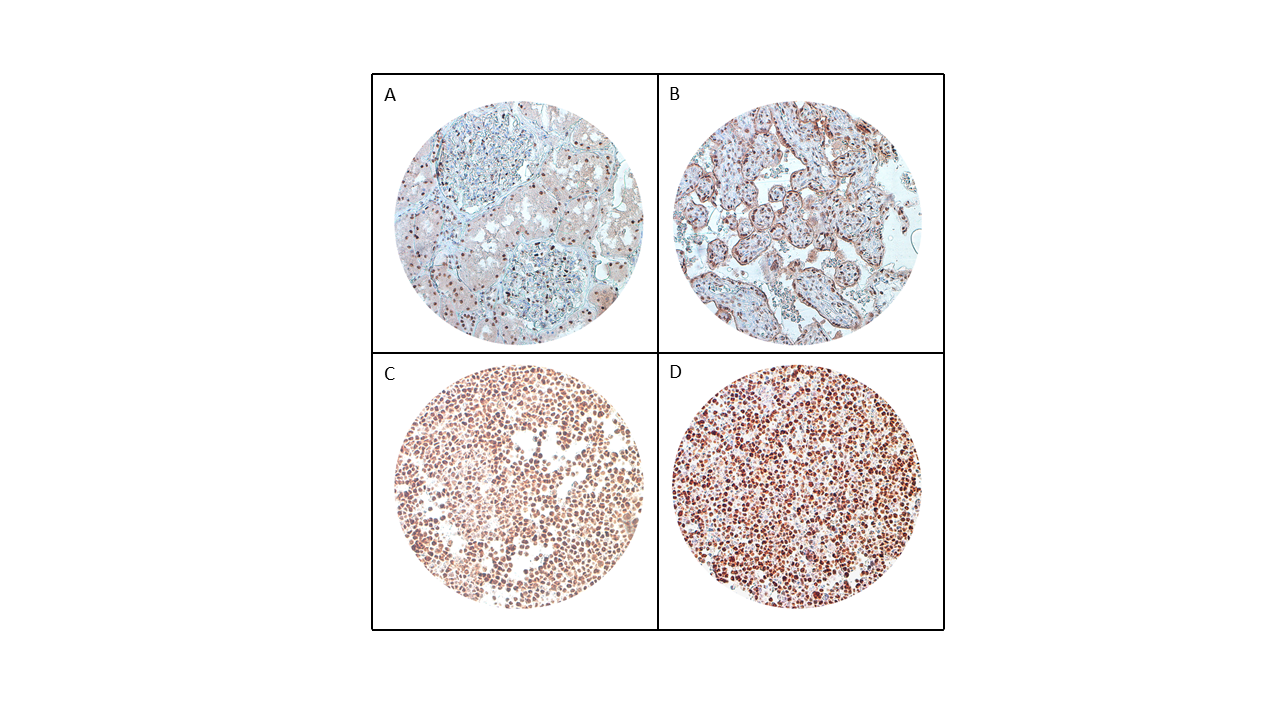

Supplement: Supplementary file 1 — Additional file 1: Figure S1. CDK9 expression in normal human tissues and ovarian cancer cell lines. CDK9 expression noted in (A) normal kidney, and (B) placenta. CDK9 high expression noted in ovarian cancer cell lines, (C) SKOV-3 and (D) OVCAR-3. [file 13048_2021_827_MOESM1_ESM.tif]
